# Expression of immune genes *RIG-I* and *Mx* in mallard ducks infected with low pathogenic avian influenza (LPAI): A dataset

**DOI:** 10.1016/j.dib.2018.04.061

**Published:** 2018-04-23

**Authors:** Anu S. Helin, Michelle Wille, Clara Atterby, Josef Järhult, Jonas Waldenström, Joanne R. Chapman

**Affiliations:** a Centre for Ecology and Evolution in Microbial Model Systems, Linnaeus University, Kalmar, Sweden; b Zoonosis Science Centre, Department of Medical Biochemistry and Microbiology, Uppsala University, Uppsala, Sweden; c Section for Infectious Diseases, Department of Medical Sciences, Uppsala University, Uppsala, Sweden; d Department of Molecular Biosciences, University of Kansas, Lawrence, USA

## Abstract

This article provides data on primer sequences used to amplify the innate immune genes *RIG-I* and *Mx* and a set of normalizing reference genes in mallards (*Anas platyrhynchos*), and shows which reference genes are stable, per tissue, for our experimental settings. Data on the expressional changes of these two genes over a time-course of infection with low pathogenic avian influenza virus (LPAI) are provided. Individual-level data are also presented, including LPAI infection load, and per tissue gene expression of *RIG-I* and *Mx*. Gene expression in two outlier individuals is explored in more depth.

**Specifications table**

**Table:** 

Subject area	*Biology*
More specific subject area	*Immunology*
Type of data	*Table, graph, figure*
How data was acquired	*Mallards were infected with low pathogenic AIV, and sacrificed over a time-course. RNA was extracted from harvested tissues and gene expression of immune genes and reference genes was analyzed via RT-qPCR on a LightCycler 480 (Roche).* Data analysis was performed using qBase+ and GraphPad Prism.
Data format	*Analyzed*
Experimental factors	*Ducks were infected with an H1N1 virus. Extracted RNA was treated with DNase.*
Experimental features	*Infection of mallards was achieved via a semi-natural, contact infection regime. qPCR results were normalized using a panel of reference genes shown to be stable for the experimental conditions under consideration.*
Data source location	*Infections were performed at the Swedish Veterinary Institute, Uppsala, Sweden. Molecular lab work was conducted at Linnaeus University, Kalmar, Sweden.*
Data accessibility	*Data are provided with this article*

**Value of the data**

• Avian influenza virus (AIV) infection of mallards was achieved via a semi-natural, contact infection route to mimic natural transmission of the virus.
• Infection with low pathogenic AIV provides a contrast to most previous studies that used highly pathogenic AIV to study immune gene expression in mallards.
• A set of reference genes that had been experimentally validated as stable under the given experimental treatment were used to stabilize RT-qPCR.
• A table summarizing the methodology and findings of previous studies of *Mx* and/or *RIG-I* expression in AIV infected ducks is provided.

## 1 Data

The dataset provided here provides additional information for Helin et al. [1]. In that paper, we show that the innate immune genes retinoic acid-inducible gene-I (*RIG-I*) and myxovirus resistance gene (*Mx*) are rapidly yet transiently upregulated after infection with low pathogenic avian influenza virus (LPAI) subtype H1N1. Helin et al. aims to provide a series of methodological improvements over previous analyses of immune gene expression in ducks infected with avian influenza virus (AIV).

Table 1 shows that most previous studies have used highly pathogenic avian influenza virus (HPAI), which is rarely detected in wild mallards [2], [3]. Additionally, infection in previous studies was achieved via artificial inoculation comprising potentially unnatural viral doses and infection routes. These previous studies have almost exclusively been conducted on domestic Pekin ducks, rather than the main wildlife reservoir for avian influenza, mallard ducks (*Anas platrhynchos*). Lastly, most previous studies have used a single, non-validated reference gene (often GAPDH) for normalizing gene expression data. This approach leads to potentially misleading interpretation of data [4].

**Table 1:** Previous studies of *RIG-I* and *Mx* gene expression in mallard and Pekin ducks infected with AIV. Only studies using quantitative real-time PCR to assess patterns of gene expression are included. Only results significantly different from controls are listed, and all fold-changes represent upregulation compared to controls (no study found down-regulation of either gene at any time point). EID_50_ is 50% egg infectious dose, MOI is multiplicity of infection, PFU is plaque forming units, RGs is reference genes, dpi is days post infection, hpi is hours post infection, N indivs is number of individuals per time point, wk is week.

**Innate Gene**	**LPAI/HPAI**	**Strain**	**Viral dose**	**Innoculation Method**	**Tissues analysed**	**RG**	**Time points**	**N. indivs**	**Breed**	**Result**^e^	**Refs.**
*RIG-I*	HPAI	H5N1	10^6^ of EID_50_	Dripped into nares, eyes & trachea	Lung, intestine	*GAPDH*	1, 3 dpi	3	Pekin	Lung: ~200-fold at 1dpi, ~20-fold at 3 dpi	[6]
										Intestine: ~5-fold at 1dpi, ~2.5-fold at 3 dpi	
*RIG-I*	LPAI	H5N2	10^6^ of EID_50_	Dripped into nares, eyes & trachea	Lung, intestine	*GAPDH*	1, 3 dpi	2-3	Pekin	No significant changes	[6]
*RIG-I*	HPAI	H5N1	10^5^ of EID_50_	Intranasal	Spleen	*β-actin*^b^	2 dpi	4	Pekin	13-fold	[7]
*RIG-I*	LPAI	H7N1	2 × 10^5^ of EID_50_	Dripped intranasally & intratracheally	Lung, bursa, ileum	*18S*^c^	0.8, 2, 4, 7, 14 dpi	6	Pekin	~7-17-fold at 0.8 dpi in all 3 tissues	[8]
*RIG-I*	HPAI	H7N1	2 × 10^5^ of EID_50_	Dripped intranasally & intratracheally	Lung, brain, spleen	*18S*	0.3, 1, 2, 3, 4, 5, 7 dpi	6	Pekin	Spleen: ~10-fold at 1&2 dpi, 2-4-fold at 3&4 dpi	[9]
										Brain: ~1.8-fold at 2 dpi	
										Lung: ~6-8-fold at 1,2&3 dpi, ~2-fold at 4 dpi	
*RIG-I*	HPAI	H5N1^a^	10^5^ of EID_50_	Intranasal	Spleen, lung	*β-actin*	2 dpi	4	Pekin	Spleen: ~65-fold in 5wk old ducks, ~4-fold in 2wk old ducks	[10]
										Lung: ~7-fold in 5wk old ducks, ~2.5-fold in 2wk old ducks	
*Mx*	LPAI	recombinant	0.1 MOI	Cells & virus mixed together	Embryo fibroblast cells	*GAPDH*	2, 4, 8, 12, 24 hpi	NA	Pekin	~500-1000-fold at 8-24 hpi	[11]
*Mx*	HPAI	H5N1	1.0 MOI	Cells & virus mixed together	Peripheral blood mononuclear cells	*GAPDH*	4, 8, 12, 24, 36, 48 hpi	NA	Mallard	25-40-fold at 8-24 hpi	[12]
*Mx*	LPAI	H1N1	0.1 MOI	Cells & virus mixed together	Primary lung cells	*GAPDH*	12, 24, 48 hpi	NA	Pekin	No significant changes	[13]
*Mx*	LPAI	H5N9	0.1 MOI	Cells & virus mixed together	Primary lung cells	*GAPDH*	12, 24, 48 hpi	NA	Pekin	~5-fold at 12 hpi, 12-fold at 24 hpi, ~8-fold at 48 hpi	[13]
*Mx*	LPAI	H7N1	10^7^ PFU	Intrachoanal cleft & oral	Illeum	*GAPDH*	1, 6 dpi	6-7	Pekin	Upregulation at 1 & 6 dpi^f^	[14]
*Mx*	LPAI	H7N1	10^7^ PFU	Intrachoanal cleft & oral	Illeum	*GAPDH*	1, 6 dpi	3^d^	Pekin	Upregulation at 1 & 6 dpi f	[15]

a Three strains, derived from chicken, egret and duck.

b Authors state β-actin was stable between uninfected and infected, but no details given and no other RGs investigated.

c Authors state that 18S had the most stable expression over time and between tissues in ducks, but data is not shown and no indication of which RGs were compared.

d Five control individuals.

e Many results were inferred from graphs because exact results were not listed. In such cases, ~ is used to indicate fold changes are approximate.

f Results not expressed as fold-change. Significant upregulation with one of the two tested viruses only.

## 2 Experimental design, materials and methods

To address these methodological issues, in Helin et al. [1] we use a semi-natural infection regime to infect mallards with low pathogenic H1N1 AIV. We then use a set of reference genes (Table 2, Table 3), that we have previously demonstrated to be stable under these experimental settings [5], to normalize RT-qPCR data. A full description of the experimental design, materials and methods is provided in Helin et al. [1].

**Table 2:** Reference genes used for each tissue type, and the number of samples available per time point per tissue.

**Tissue**	**RGs**	**Number of samples/time point**					
		**0 dpi**	**0.5 dpi**	**1 dpi**	**2 dpi**	**4 dpi**	**7dpi**
Blood	RPS13, UBE20, RPL4	5	5	5	5	5	5
Spleen	RPS13, SDHA, GAPDH	5	5	5	5	5	5
GI1	RPS13, RPL4	4	4	4	4	5	4
GI2	RPL4, RPL30	4	3	4	5	5	4
Colon	RPL4, SDHA	5	4	3	4	5	3

**Table 3:** Primers used in [1]. F denotes the forward primer and R the reverse primer. Annealing temperature (Ta) expressed in °C and length in base pairs (bp).

**Gene Symbol**	**Gene Name**		**Primers**	**Ta**	**Length**
*RIG-I*	Retinoic acid-inducible gene-I	F	GTGTATGGAGGAAAACCCTATTCTTAACT	59	95
		R	GGAGGGGTGATACCTGTTGTTTGAT		
*Mx*	Myxovirus resistance	F	TTCATGACTTCGGCGACAAC	59	128
		R	AACTCGGCCACTGAGGTAAT		
*GAPDH*	Glyceraldehyde-3-phosphate dehydrogenase	F	GGTTGTCTCCTGCGACTTCA	60	164
		R	TCCTTGGATGCCATGTGGAC		
*RPL4*	Ribosomal protein L4	F	CCTGGGCCTTAGCTGTAACC	60	115
		R	AAGCTGAACCCATACGCCAA		
*RPL30*	Ribosomal protein L30	F	CTCAATGTTGTTGCCGCTGT	60	119
		R	GCAAAGCCAAGCTGGTCATC		
*RPS13*	Ribosomal protein S13	F	AAGAAAGGCCTGACTCCCTC	59	82
		R	TGCCAGTAACAAAGCGAACC		
*SDHA*	Succinate dehydrogenase complex, subunit A	F	GACACAGTGAAAGGCTCCGA	60	90
		R	CTCCAGCTCTATCACGGCAG		
*UBE20*	Ubiquitin-conjugating enzyme E2O	F	AGCATCCCCCTTTCCATCAA	59	91
		R	CAACCCTGTCTCCTGGCTTA		

Datasets describing the fold-change in expression between experimental time-points, and per individual, for each tissue type and gene are provided as Supplementary tables S1–4 and Figs. S1–S4 to this article. Fig. S5 provides a more in-depth analysis of two individuals with extremely high expression, showing that this over-expression was restricted to a specific tissue and a single gene at single time-point.

## References

[1] Helin A.S., Wille M., Atterby C., Järhult J. (2018). A rapid and transient innate immune response to avian influenza infection in mallards. Mol. Immunol..

[2] Latorre-Margalef N., Tolf C., Grosbois V., Avril A. (2014). Long-term variation in influenza A virus prevalence and subtype diversity in migratory mallards in northern Europe. Proc. R. Soc. Lond. B Biol. Sci..

[3] Olson S.H., Parmley J., Soos C., Gilbert M. (2014). Sampling strategies and biodiversity of influenza A subtypes in wild birds. PLoS One.

[4] Chapman J.R., Waldenström J. (2015). With reference to reference genes: a systematic review of endogenous controls in gene expression studies. PLoS One.

[5] Chapman J.R., Helin A.S., Wille M., Atterby C. (2016). A panel of stably expressed reference genes for real-time qPCR gene expression studies of mallards (*Anas**platyrhynchos*). PLoS One.

[6] Barber M.R., Aldridge J.R., Webster R.G., Magor K.E. (2010). Association of RIG-I with innate immunity of ducks to influenza. Proc. Natl. Acad. Sci. USA.

[7] Cagle C., Wasilenko J., Adams S.C., Cardona C.J. (2012). Differences in pathogenicity, response to vaccination, and innate immune responses in different types of ducks infected with a virulent H5N1 highly pathogenic avian influenza virus from Vietnam. Avian Dis..

[8] Cornelissen J.B., Post J., Peeters B., Vervelde L., Rebel J.M. (2012). Differential innate responses of chickens and ducks to low-pathogenic avian influenza. Avian Pathol..

[9] Cornelissen J.B., Vervelde L., Post J., Rebel J.M. (2013). Differences in highly pathogenic avian influenza viral pathogenesis and associated early inflammatory response in chickens and ducks. Avian Pathol..

[10] Pantin-Jackwood M.J., Smith D.M., Wasilenko J.L., Cagle C. (2012). Effect of age on the pathogenesis and innate immune responses in Pekin ducks infected with different H5N1 highly pathogenic avian influenza viruses. Virus Res..

[11] Adams S., Xing Z., Li J.L., Mendoza K. (2013). The effect of avian influenza virus NS1 allele on virus replication and innate gene expression in avian cells. Mol. Immunol..

[12] Cui Z., Hu J., He L., Li Q. (2014). Differential immune response of mallard duck peripheral blood mononuclear cells to two highly pathogenic avian influenza H5N1 viruses with distinct pathogenicity in mallard ducks. Arch. Virol..

[13] Jiang H., Yang H., Kapczynski D.R. (2011). Chicken interferon alpha pretreatment reduces virus replication of pandemic H1N1 and H5N9 avian influenza viruses in lung cell cultures from different avian species. Virol. J..

[14] Soubies S.M., Volmer C., Croville G., Loupias J. (2010). Species-specific contribution of the four C-terminal amino acids of influenza A virus NS1 protein to virulence. J. Virol..

[15] Volmer C., Soubies S.M., Grenier B., Guerin J.L., Volmer R. (2011). Immune response in the duck intestine following infection with low-pathogenic avian influenza viruses or stimulation with a Toll-like receptor 7 agonist administered orally. J. Gen. Virol..

