# Supplementary material for: Expression of immune genes RIG-I and Mx in mallard ducks infected with low pathogenic avian influenza (LPAI): A dataset
Source: Data Brief. 2018 Apr 23;18:1562–6. doi: 10.1016/j.dib.2018.04.061 (PMC5998173; doi:10.1016/j.dib.2018.04.061)
Supplement: Supplementary file 1 — Supplementary material [file mmc1.docx]

Conflict of interest

All the author confirms no conflict of interest.
