# Supplementary material for: Expression of immune genes RIG-I and Mx in mallard ducks infected with low pathogenic avian influenza (LPAI): A dataset
Source: Data Brief. 2018 Apr 23;18:1562–6. doi: 10.1016/j.dib.2018.04.061 (PMC5998173; doi:10.1016/j.dib.2018.04.061)
Supplement: Supplementary file 2 — Supplementary material [file mmc2.docx]

**Fig S1.** AIV infection load, as measured by AIV-specific qPCR, in individuals sampled (A) 0.5 days post infection (dpi); (B) one dpi; and (C) two dpi. In all cases, individuals are ordered by the time-point at which they were subsequently sacrificed. Samples 6 - 10 are absent from panels B and C because they were sacrificed at 0.5 dpi, immediately after being sampled for AIV infection. Likewise, samples 11 – 15 are absent from panel C because they were sacrificed at 1 dpi.

**
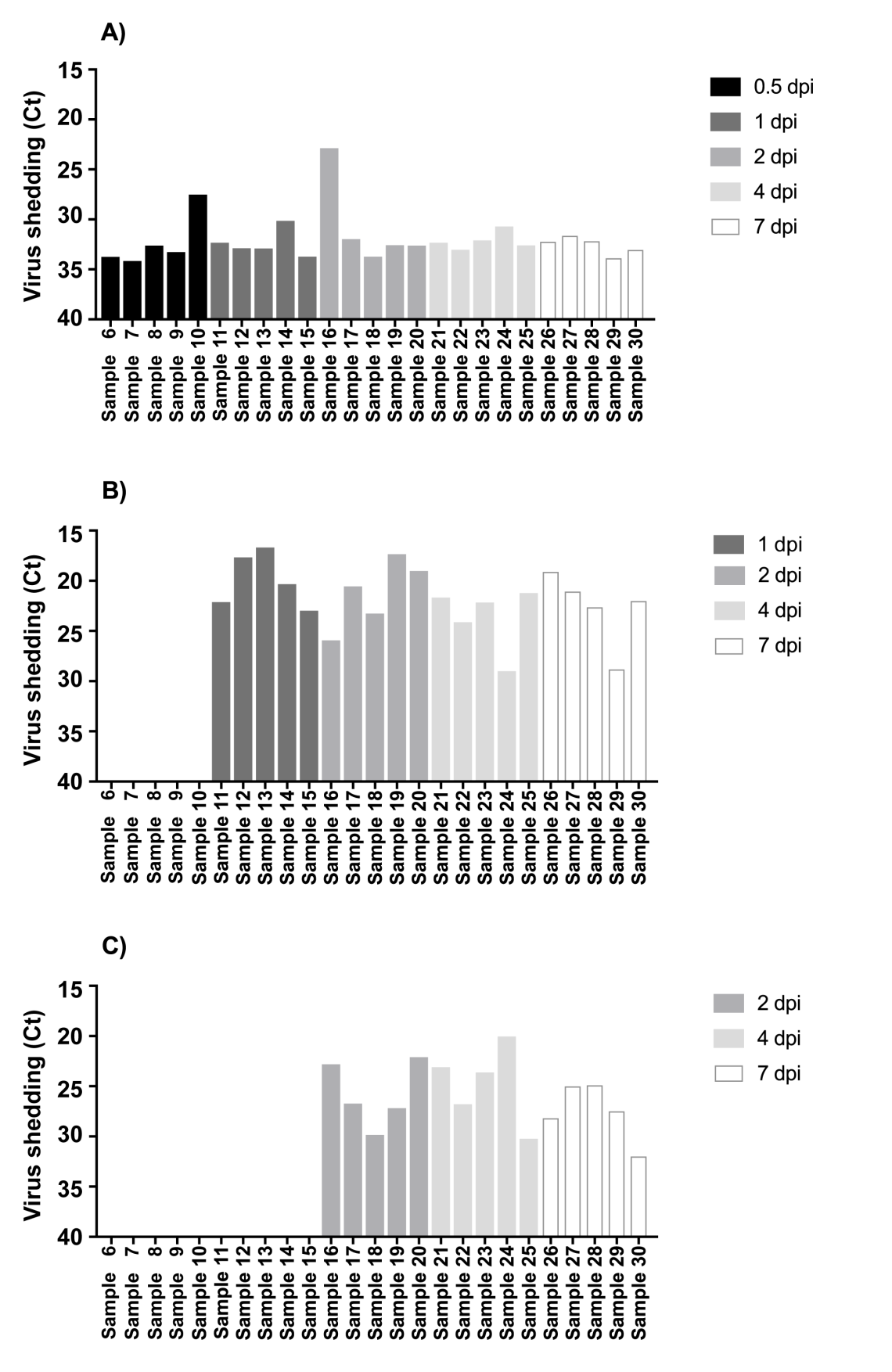
**

**Figure S2.** Expression of *RIG-I* in all individuals, ordered by time point. Samples 1-5 are uninfected controls, samples 6-10 are 0.5 dpi, samples 11-15 are 1 dpi, samples 15-20 are 2 dpi, samples 21-25 are 4 dpi and samples 26-30 are 7 dpi.

**
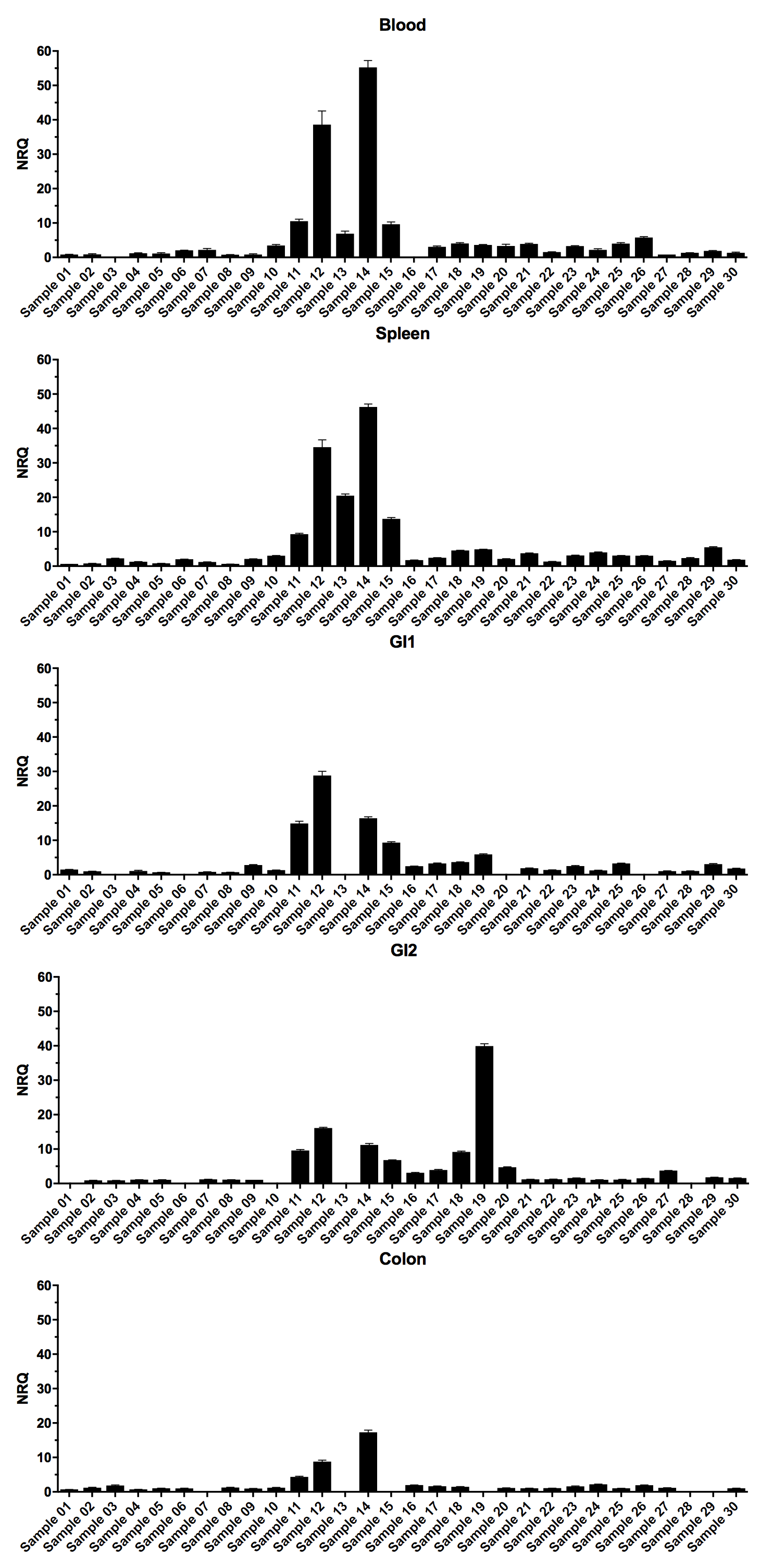
**

**Fig S3.** Normalized Relative Quantity (NRQ) values for (A) *RIG-I* and (B) *Mx* across all individuals, per tissue type. Individuals are ordered by time point, such that the first five bars represent 0.5 dpi individuals, the next five bars are 1 dpi individuals and so forth.


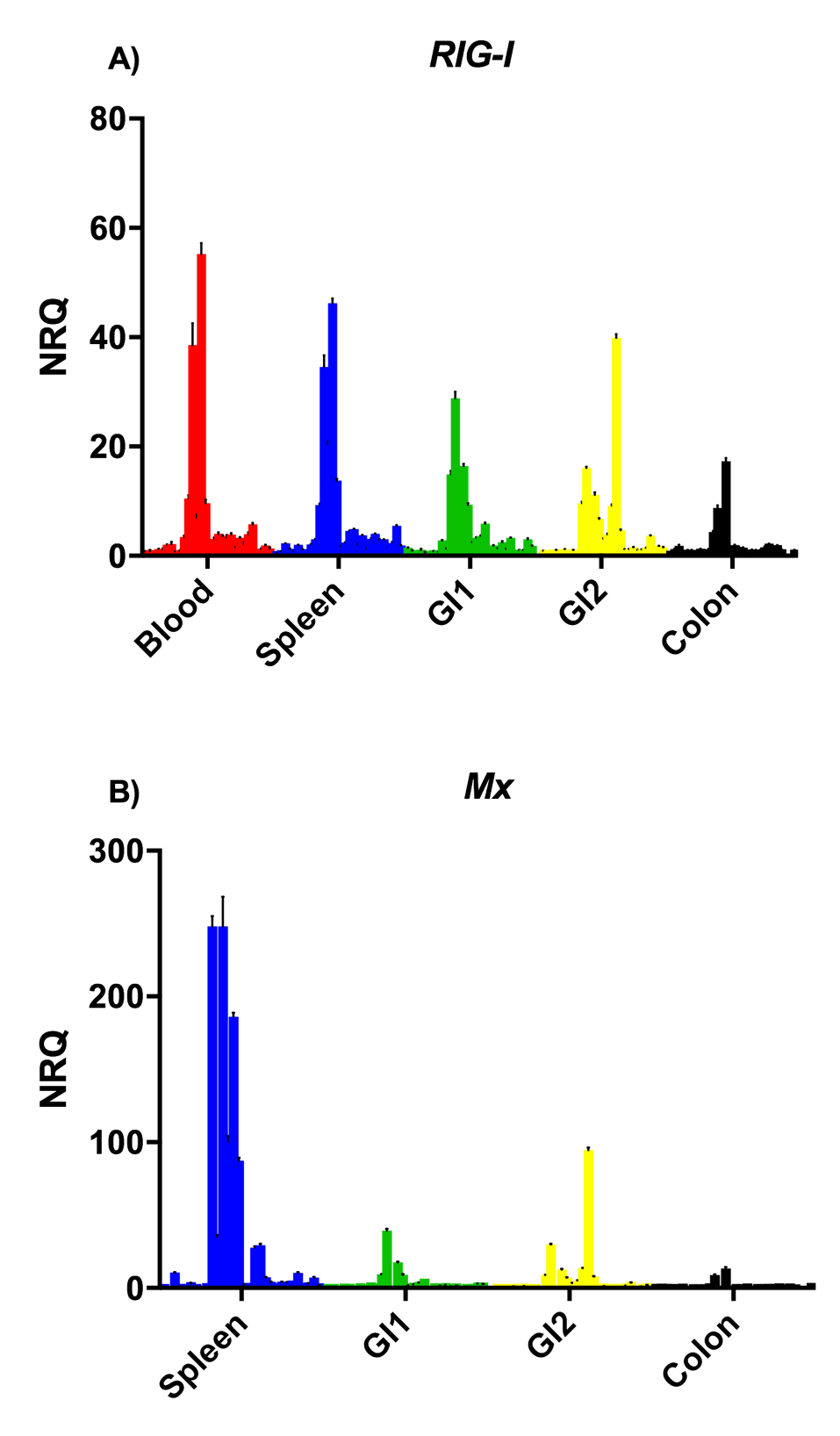


**Figure S4.** Expression of *Mx* in all individuals, ordered by time point. Samples 1-5 are uninfected controls, samples 6-10 are 0.5 dpi, samples 11-15 are 1 dpi, samples 15-20 are 2 dpi, samples 21-25 are 4 dpi and samples 26-30 are 7 dpi.

**
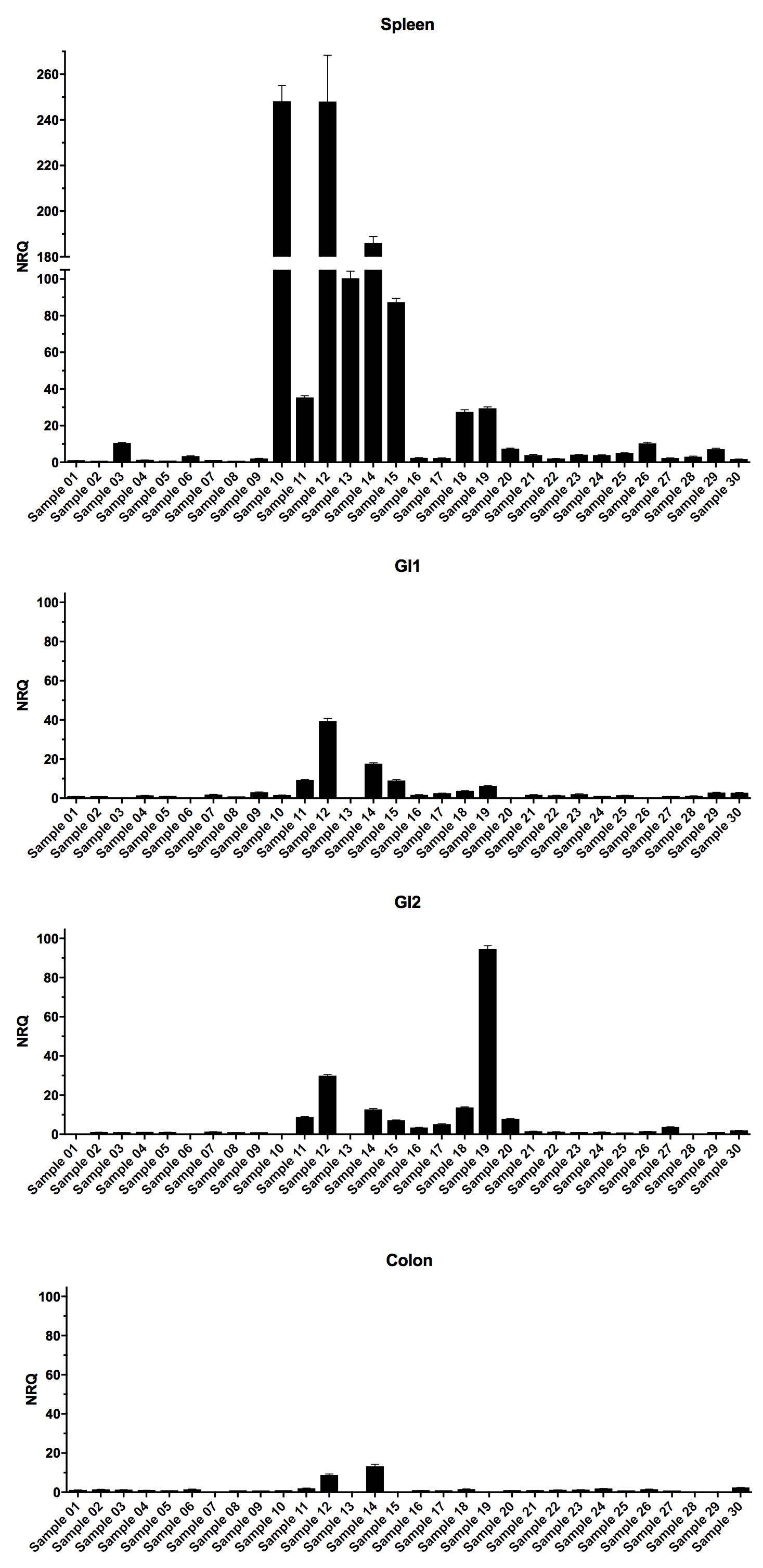
**

**Fig S5.** Expression of *RIG-I* (black bars) and *Mx* (grey bars) in four tissues taken from (A) sample 10 at 0.5 dpi and (B) sample 19 at 2 dpi. Note that for sample 10, GI2 data is missing and for sample 19, colon data is missing, as these samples were lost in transit. Additionally, there is no data for *Mx* in blood for either individual, as *Mx* was not detectable in peripheral blood in any individuals.

**
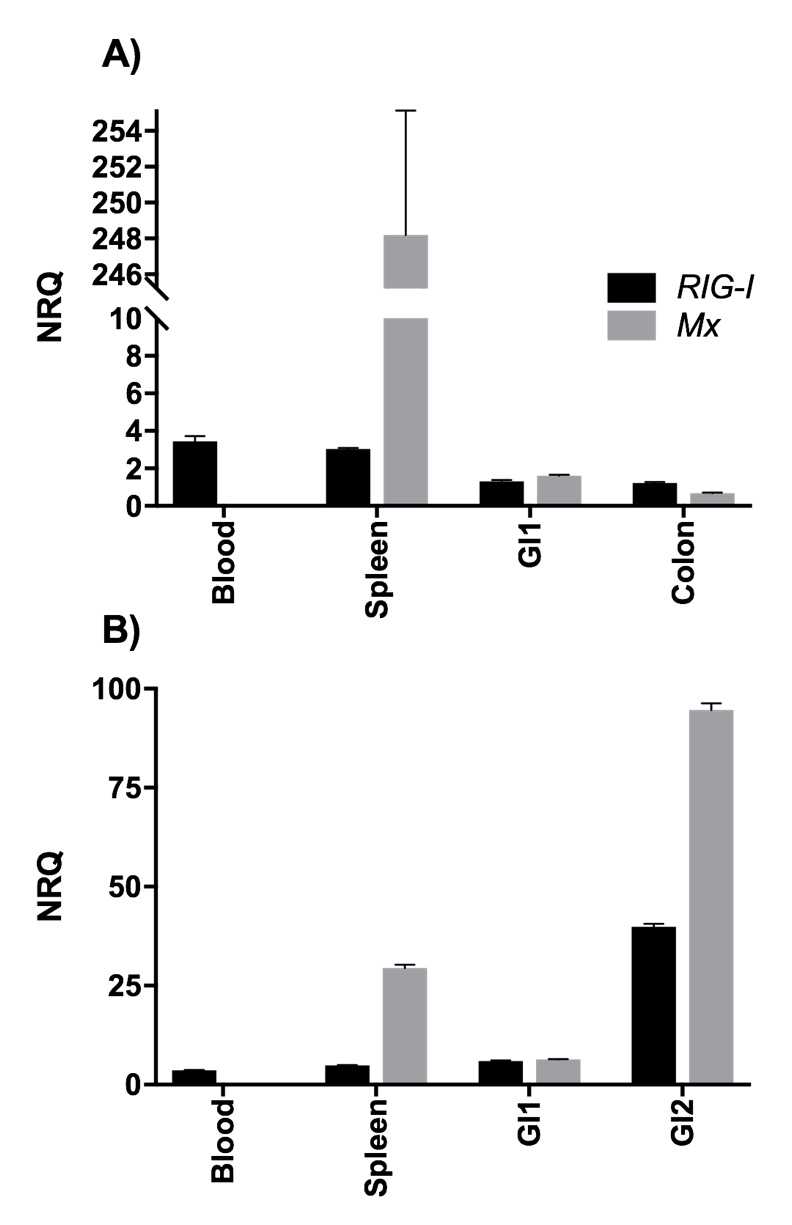
**
