# Supplementary material for: Expression of immune genes RIG-I and Mx in mallard ducks infected with low pathogenic avian influenza (LPAI): A dataset
Source: Data Brief. 2018 Apr 23;18:1562–6. doi: 10.1016/j.dib.2018.04.061 (PMC5998173; doi:10.1016/j.dib.2018.04.061)
Supplement: Supplementary file 3 — Supplementary material [file mmc3.docx]

**Table S1.** Fold-change comparisons between the different time-points for *RIG-I* expression in (a) blood, (b) spleen, (c) GI1, (d) GI2, and (e) colon. CI is confidence interval. Significant P-values (P<0.05) are shown in bold.

| **Comparison** | **Fold-change ratio** | **95% lower CI** | **95% upper CI** | **P-value** |
| --- | --- | --- | --- | --- |
| *(a) BLOOD* |  |  |  |  |
| 0.5 dpi vs. control | 1.586 | 0.447 | 5.623 | 0.475 |
| 1 dpi vs. control | 17.167 | 4.842 | 60.867 | **<0.0001** |
| 2 dpi vs. control | 3.488 | 0.919 | 13.242 | 0.066 |
| 4 dpi vs. control | 2.799 | 0.789 | 9.925 | 0.111 |
| 7 dpi vs. control | 1.737 | 0.49 | 6.158 | 0.393 |
| 0.5 dpi vs. 1 dpi | 0.092 | 0.028 | 0.305 | **<0.0001** |
| 0.5 dpi vs. 2 dpi | 0.455 | 0.128 | 1.612 | 0.223 |
| 0.5 dpi vs. 4 dpi | 0.567 | 0.172 | 1.868 | 0.351 |
| 1 dpi vs. 2 dpi | 4.922 | 1.388 | 17.453 | **0.014** |
| 1 dpi vs. 4 dpi | 6.133 | 1.86 | 20.226 | **0.003** |
| 2 dpi vs. 4 dpi | 1.246 | 0.351 | 4.418 | 0.734 |
| 7 dpi vs. 0.5 dpi | 1.095 | 0.332 | 3.612 | 0.882 |
| 7 dpi vs. 1 dpi | 0.101 | 0.031 | 0.334 | **0.0002** |
| 7 dpi vs. 2 dpi | 0.498 | 0.14 | 1.766 | 0.281 |
| 7 dpi vs. 4 dpi | 0.62 | 0.188 | 2.046 | 0.433 |
| *(b) SPLEEN* |  |  |  |  |
| 0.5 dpi vs. control | 1.552 | 0.53 | 4.543 | 0.423 |
| 1 dpi vs. control | 21.093 | 7.208 | 61.722 | **<0.0001** |
| 2 dpi vs. control | 2.88 | 0.984 | 8.428 | 0.054 |
| 4 dpi vs. control | 2.866 | 0.979 | 8.387 | 0.055 |
| 7 dpi vs. control | 2.57 | 0.878 | 7.522 | 0.085 |
| 0.5 dpi vs. 1 dpi | 0.074 | 0.025 | 0.215 | **<0.0001** |
| 0.5 dpi vs. 2 dpi | 0.539 | 0.184 | 1.577 | 0.259 |
| 0.5 dpi vs. 4 dpi | 0.542 | 0.185 | 1.585 | 0.264 |
| 1 dpi vs. 2 dpi | 7.323 | 2.503 | 21.429 | **0.0003** |
| 1 dpi vs. 4 dpi | 7.359 | 2.515 | 21.535 | **0.0003** |
| 2 dpi vs. 4 dpi | 1.005 | 0.343 | 2.941 | 0.993 |
| 7 dpi vs. 0.5 dpi | 1.656 | 0.566 | 4.845 | 0.357 |
| 7 dpi vs. 1 dpi | 0.122 | 0.042 | 0.357 | **0.0001** |
| 7 dpi vs. 2 dpi | 0.892 | 0.305 | 2.611 | 0.835 |
| 7 dpi vs. 4 dpi | 0.897 | 0.306 | 2.624 | 0.843 |
| *(c) GI1* |  |  |  |  |
| 0.5 dpi vs. control | 1.213 | 0.437 | 3.368 | 0.711 |
| 1 dpi vs. control | 16.012 | 5.768 | 44.446 | **<0.0001** |
| 2 dpi vs. control | 3.638 | 1.311 | 10.098 | **0.013** |
| 4 dpi vs. control | 1.933 | 0.734 | 5.093 | 0.182 |
| 7 dpi vs. control | 1.586 | 0.571 | 4.403 | 0.376 |
| 0.5 dpi vs. 1 dpi | 0.076 | 0.027 | 0.21 | **<0.0001** |
| 0.5 dpi vs. 2 dpi | 0.334 | 0.12 | 0.926 | **0.035** |
| 0.5 dpi vs. 4 dpi | 0.628 | 0.238 | 1.653 | 0.347 |
| 1 dpi vs. 2 dpi | 4.401 | 1.586 | 12.217 | **0.004** |
| 1 dpi vs. 4 dpi | 8.281 | 3.144 | 21.814 | **<0.0001** |
| 2 dpi vs. 4 dpi | 1.882 | 0.714 | 4.956 | 0.201 |
| 7 dpi vs. 0.5 dpi | 1.307 | 0.471 | 3.629 | 0.607 |
| 7 dpi vs. 1 dpi | 0.099 | 0.036 | 0.275 | **<0.0001** |
| 7 dpi vs. 2 dpi | 0.436 | 0.157 | 1.21 | 0.111 |
| 7 dpi vs. 4 dpi | 0.82 | 0.311 | 2.161 | 0.688 |
| *(d) GI2* |  |  |  |  |
| 0.5 dpi vs. control | 1.127 | 0.315 | 4.024 | 0.854 |
| 1 dpi vs. control | 10.409 | 3.203 | 33.826 | **<0.0001** |
| 2 dpi vs. control | 7.333 | 2.397 | 22.432 | **0.0005** |
| 4 dpi vs. control | 1.248 | 0.408 | 3.818 | 0.698 |
| 7 dpi vs. control | 1.998 | 0.615 | 6.493 | 0.250 |
| 0.5 dpi vs. 1 dpi | 0.108 | 0.03 | 0.387 | **0.0006** |
| 0.5 dpi vs. 2 dpi | 0.154 | 0.045 | 0.519 | **0.003** |
| 0.5 dpi vs. 4 dpi | 0.903 | 0.267 | 3.049 | 0.870 |
| 1 dpi vs. 2 dpi | 1.419 | 0.464 | 4.342 | 0.540 |
| 1 dpi vs. 4 dpi | 8.34 | 2.726 | 25.51 | **0.0002** |
| 2 dpi vs. 4 dpi | 5.875 | 2.048 | 16.859 | **0.001** |
| 7 dpi vs. 0.5 dpi | 1.773 | 0.497 | 6.334 | 0.378 |
| 7 dpi vs. 1 dpi | 0.192 | 0.059 | 0.624 | **0.006** |
| 7 dpi vs. 2 dpi | 0.272 | 0.089 | 0.833 | **0.023** |
| 7 dpi vs. 4 dpi | 1.601 | 0.523 | 4.897 | 0.410 |
| *(e) COLON* |  |  |  |  |
| 0.5 dpi vs. control | 1.096 | 0.484 | 2.48 | 0.826 |
| 1 dpi vs. control | 8.709 | 3.578 | 21.196 | **<0.0001** |
| 2 dpi vs. control | 1.524 | 0.673 | 3.449 | 0.312 |
| 4 dpi vs. control | 1.319 | 0.61 | 2.849 | 0.481 |
| 7 dpi vs. control | 1.329 | 0.546 | 3.234 | 0.531 |
| 0.5 dpi vs. 1 dpi | 0.126 | 0.05 | 0.319 | **<0.0001** |
| 0.5 dpi vs. 2 dpi | 0.719 | 0.304 | 1.701 | 0.453 |
| 0.5 dpi vs. 4 dpi | 0.831 | 0.367 | 1.881 | 0.657 |
| 1 dpi vs. 2 dpi | 5.715 | 2.254 | 14.49 | **0.0002** |
| 1 dpi vs. 4 dpi | 6.604 | 2.713 | 16.075 | **<0.0001** |
| 2 dpi vs. 4 dpi | 1.156 | 0.51 | 2.616 | 0.728 |
| 7 dpi vs. 0.5 dpi | 1.213 | 0.478 | 3.075 | 0.684 |
| 7 dpi vs. 1 dpi | 0.153 | 0.056 | 0.413 | **0.0002** |
| 7 dpi vs. 2 dpi | 0.872 | 0.344 | 2.211 | 0.773 |
| 7 dpi vs. 4 dpi | 1.008 | 0.414 | 2.453 | 0.986 |

* Significance (*P* < 0.05) tested with one-way ANOVA followed by Tukey-Kramer correction for multiple testing.
