# Supplementary material for: Expression of immune genes RIG-I and Mx in mallard ducks infected with low pathogenic avian influenza (LPAI): A dataset
Source: Data Brief. 2018 Apr 23;18:1562–6. doi: 10.1016/j.dib.2018.04.061 (PMC5998173; doi:10.1016/j.dib.2018.04.061)
Supplement: Supplementary file 4 — Supplementary material [file mmc4.docx]

**Table S2.** *RIG-I* results per individual per tissue. Results are expressed as Normalized Relative Quantity (NRQ) and NRQ standard error (SE). Blanks occur when a particular tissue was not available for an individual due to labels peeling off tubes during transport in liquid nitrogen.

|  | **Blood** | | **Spleen** | | | **GI1** | | | **GI2** | | | **Colon** | | |  |
| --- | --- | --- | --- | --- | --- | --- | --- | --- | --- | --- | --- | --- | --- | --- | --- |
|  | **NRQ** | **SE** | | **NRQ** | **SE** | | **NRQ** | **SE** | | **NRQ** | **SE** | | **NRQ** | **SE** | |
| **Sample 01** | 0.839 | 0.053 | | 0.522 | 0.023 | | 1.488 | 0.065 | |  |  | | 0.647 | 0.065 | |
| **Sample 02** | 0.871 | 0.206 | | 0.804 | 0.031 | | 0.986 | 0.041 | | 0.928 | 0.020 | | 1.221 | 0.101 | |
| **Sample 03** |  |  | | 2.282 | 0.045 | |  |  | | 0.895 | 0.017 | | 1.834 | 0.185 | |
| **Sample 04** | 1.206 | 0.117 | | 1.295 | 0.039 | | 1.065 | 0.225 | | 1.111 | 0.018 | | 0.665 | 0.065 | |
| **Sample 05** | 1.135 | 0.229 | | 0.806 | 0.037 | | 0.640 | 0.066 | | 1.084 | 0.040 | | 1.039 | 0.047 | |
| **Sample 06** | 2.045 | 0.051 | | 2.020 | 0.038 | |  |  | |  |  | | 0.994 | 0.077 | |
| **Sample 07** | 2.170 | 0.414 | | 1.213 | 0.037 | | 0.828 | 0.043 | | 1.206 | 0.027 | |  |  | |
| **Sample 08** | 0.792 | 0.045 | | 0.578 | 0.019 | | 0.704 | 0.056 | | 1.102 | 0.019 | | 1.268 | 0.051 | |
| **Sample 09** | 0.830 | 0.194 | | 2.093 | 0.057 | | 2.819 | 0.105 | | 1.077 | 0.012 | | 0.933 | 0.048 | |
| **Sample 10** | 3.440 | 0.290 | | 3.038 | 0.057 | | 1.320 | 0.064 | |  |  | | 1.225 | 0.054 | |
| **Sample 11** | 10.524 | 0.593 | | 9.282 | 0.270 | | 14.879 | 0.689 | | 9.601 | 0.294 | | 4.341 | 0.201 | |
| **Sample 12** | 38.586 | 3.983 | | 34.589 | 2.101 | | 28.811 | 1.239 | | 16.109 | 0.221 | | 8.793 | 0.427 | |
| **Sample 13** | 6.890 | 0.756 | | 20.456 | 0.530 | |  |  | |  |  | |  |  | |
| **Sample 14** | 55.245 | 1.970 | | 46.241 | 0.885 | | 16.414 | 0.425 | | 11.195 | 0.446 | | 17.304 | 0.616 | |
| **Sample 15** | 9.646 | 0.684 | | 13.748 | 0.366 | | 9.341 | 0.263 | | 6.781 | 0.101 | |  |  | |
| **Sample 16** |  |  | | 1.764 | 0.042 | | 2.460 | 0.080 | | 3.120 | 0.115 | | 1.986 | 0.043 | |
| **Sample 17** | 3.078 | 0.262 | | 2.423 | 0.051 | | 3.291 | 0.129 | | 3.926 | 0.159 | | 1.637 | 0.067 | |
| **Sample 18** | 4.031 | 0.284 | | 4.556 | 0.074 | | 3.652 | 0.095 | | 9.178 | 0.251 | | 1.471 | 0.064 | |
| **Sample 19** | 3.602 | 0.137 | | 4.872 | 0.064 | | 5.925 | 0.168 | | 39.895 | 0.702 | |  |  | |
| **Sample 20** | 3.310 | 0.528 | | 2.090 | 0.088 | |  |  | | 4.729 | 0.114 | | 1.127 | 0.043 | |
| **Sample 21** | 3.924 | 0.221 | | 3.761 | 0.072 | | 1.884 | 0.102 | | 1.214 | 0.017 | | 1.041 | 0.036 | |
| **Sample 22** | 1.540 | 0.089 | | 1.340 | 0.023 | | 1.384 | 0.019 | | 1.264 | 0.018 | | 1.060 | 0.038 | |
| **Sample 23** | 3.284 | 0.121 | | 3.114 | 0.093 | | 2.517 | 0.166 | | 1.595 | 0.031 | | 1.596 | 0.106 | |
| **Sample 24** | 2.171 | 0.368 | | 4.019 | 0.096 | | 1.257 | 0.092 | | 1.090 | 0.038 | | 2.201 | 0.045 | |
| **Sample 25** | 3.988 | 0.303 | | 3.067 | 0.040 | | 3.276 | 0.111 | | 1.135 | 0.074 | | 1.028 | 0.021 | |
| **Sample 26** | 5.780 | 0.264 | | 3.017 | 0.041 | |  |  | | 1.489 | 0.029 | | 1.947 | 0.049 | |
| **Sample 27** | 0.822 | 0.017 | | 1.531 | 0.059 | | 1.049 | 0.054 | | 3.732 | 0.068 | | 1.150 | 0.041 | |
| **Sample 28** | 1.325 | 0.052 | | 2.366 | 0.175 | | 1.091 | 0.036 | |  |  | |  |  | |
| **Sample 29** | 1.882 | 0.127 | | 5.477 | 0.158 | | 3.056 | 0.194 | | 1.806 | 0.041 | |  |  | |
| **Sample 30** | 1.335 | 0.229 | | 1.875 | 0.059 | | 1.811 | 0.084 | | 1.587 | 0.048 | | 1.048 | 0.033 | |
