# Supplementary material for: Expression of immune genes RIG-I and Mx in mallard ducks infected with low pathogenic avian influenza (LPAI): A dataset
Source: Data Brief. 2018 Apr 23;18:1562–6. doi: 10.1016/j.dib.2018.04.061 (PMC5998173; doi:10.1016/j.dib.2018.04.061)
Supplement: Supplementary file 5 — Supplementary material [file mmc5.docx]

**Table S3.** Fold-change comparisons between the different time-points for *Mx* expression in (a) spleen, (b) GI1, (c) GI2, and (d) colon. CI is confidence interval. Significant P-values (P<0.05) are shown in bold.

| **Comparison** | **Fold-change ratio** | **95% lower CI** | **95% upper CI** | **P-value** |
| --- | --- | --- | --- | --- |
| *(a) SPLEEN* |  |  |  |  |
| 0.5 dpi vs. control | 3.298 | 0.214 | 50.907 | 0.393 |
| 1 dpi vs. control | 107.486 | 6.964 | 1 658.92 | **0.001** |
| 2 dpi vs. control | 8.09 | 0.524 | 124.867 | 0.134 |
| 4 dpi vs. control | 3.694 | 0.239 | 57.01 | 0.349 |
| 7 dpi vs. control | 3.932 | 0.255 | 60.689 | 0.327 |
| 0.5 dpi vs. 1 dpi | 0.031 | 0.002 | 0.474 | **0.013** |
| 0.5 dpi vs. 2 dpi | 0.408 | 0.026 | 6.292 | 0.522 |
| 0.5 dpi vs. 4 dpi | 0.893 | 0.058 | 13.782 | 0.935 |
| 1 dpi vs. 2 dpi | 13.285 | 0.861 | 205.046 | 0.064 |
| 1 dpi vs. 4 dpi | 29.099 | 1.885 | 449.107 | **0.016** |
| 2 dpi vs. 4 dpi | 2.19 | 0.142 | 33.804 | 0.574 |
| 7 dpi vs. 0.5 dpi | 1.192 | 0.077 | 18.399 | 0.900 |
| 7 dpi vs. 1 dpi | 0.037 | 0.002 | 0.565 | **0.022** |
| 7 dpi vs. 2 dpi | 0.486 | 0.031 | 7.501 | 0.606 |
| 7 dpi vs. 4 dpi | 1.065 | 0.069 | 16.43 | 0.964 |
| *(b) GI1* |  |  |  |  |
| 0.5 dpi vs. control | 1.466 | 0.427 | 5.036 | 0.543 |
| 1 dpi vs. control | 15.56 | 4.529 | 53.456 | **<0.0001** |
| 2 dpi vs. control | 3.205 | 0.933 | 11.01 | 0.064 |
| 4 dpi vs. control | 1.519 | 0.471 | 4.899 | 0.484 |
| 7 dpi vs. control | 1.72 | 0.501 | 5.91 | 0.389 |
| 0.5 dpi vs. 1 dpi | 0.094 | 0.027 | 0.324 | **0.0002** |
| 0.5 dpi vs. 2 dpi | 0.457 | 0.133 | 1.572 | 0.214 |
| 0.5 dpi vs. 4 dpi | 0.965 | 0.299 | 3.112 | 0.953 |
| 1 dpi vs. 2 dpi | 4.855 | 1.413 | 16.68 | **0.012** |
| 1 dpi vs. 4 dpi | 10.243 | 3.176 | 33.028 | **<0.0001** |
| 2 dpi vs. 4 dpi | 3.205 | 0.933 | 11.01 | 0.064 |
| 7 dpi vs. 0.5 dpi | 1.174 | 0.342 | 4.032 | 0.799 |
| 7 dpi vs. 1 dpi | 0.111 | 0.032 | 0.38 | **0.001** |
| 7 dpi vs. 2 dpi | 0.537 | 0.156 | 1.844 | 0.324 |
| 7 dpi vs. 4 dpi | 1.132 | 0.351 | 3.652 | 0.836 |
| *(c) GI2* |  |  |  |  |
| 0.5 dpi vs. control | 0.952 | 0.164 | 5.534 | 0.956 |
| 1 dpi vs. control | 12.501 | 2.45 | 63.782 | **0.002** |
| 2 dpi vs. control | 11.259 | 2.399 | 52.835 | **0.002** |
| 4 dpi vs. control | 0.959 | 0.204 | 4.503 | 0.958 |
| 7 dpi vs. control | 1.812 | 0.355 | 9.246 | 0.475 |
| 0.5 dpi vs. 1 dpi | 0.076 | 0.013 | 0.443 | **0.004** |
| 0.5 dpi vs. 2 dpi | 0.085 | 0.016 | 0.455 | **0.004** |
| 0.5 dpi vs. 4 dpi | 0.992 | 0.184 | 5.34 | 0.993 |
| 1 dpi vs. 2 dpi | 1.11 | 0.237 | 5.211 | 0.895 |
| 1 dpi vs. 4 dpi | 13.029 | 2.776 | 61.143 | **0.001** |
| 2 dpi vs. 4 dpi | 11.734 | 2.732 | 50.407 | **0.001** |
| 7 dpi vs. 0.5 dpi | 1.904 | 0.327 | 11.067 | 0.474 |
| 7 dpi vs. 1 dpi | 0.145 | 0.028 | 0.74 | **0.021** |
| 7 dpi vs. 2 dpi | 0.161 | 0.034 | 0.755 | **0.021** |
| 7 dpi vs. 4 dpi | 1.889 | 0.402 | 8.864 | 0.420 |
| *(d) COLON* |  |  |  |  |
| 0.5 dpi vs. control | 0.681 | 0.183 | 2.532 | 0.567 |
| 1 dpi vs. control | 6.173 | 1.478 | 25.791 | **0.013** |
| 2 dpi vs. control | 0.887 | 0.238 | 3.297 | 0.858 |
| 4 dpi vs. control | 0.979 | 0.284 | 3.376 | 0.973 |
| 7 dpi vs. control | 1.113 | 0.266 | 4.651 | 0.883 |
| 0.5 dpi vs. 1 dpi | 0.11 | 0.025 | 0.492 | **0.004** |
| 0.5 dpi vs. 2 dpi | 0.768 | 0.192 | 3.066 | 0.709 |
| 0.5 dpi vs. 4 dpi | 0.696 | 0.187 | 2.587 | 0.589 |
| 1 dpi vs. 2 dpi | 6.962 | 1.561 | 31.058 | **0.011** |
| 1 dpi vs. 4 dpi | 6.307 | 1.51 | 26.351 | **0.012** |
| 2 dpi vs. 4 dpi | 0.906 | 0.244 | 3.369 | 0.883 |
| 7 dpi vs. 0.5 dpi | 1.635 | 0.367 | 7.293 | 0.519 |
| 7 dpi vs. 1 dpi | 0.18 | 0.036 | 0.892 | **0.036** |
| 7 dpi vs. 2 dpi | 1.256 | 0.281 | 5.601 | 0.765 |
| 7 dpi vs. 4 dpi | 1.138 | 0.272 | 4.752 | 0.859 |

* Significance (*P* < 0.05) tested with one-way ANOVA followed by Tukey-Kramer correction for multiple testing.
