# Supplementary material for: Expression of immune genes RIG-I and Mx in mallard ducks infected with low pathogenic avian influenza (LPAI): A dataset
Source: Data Brief. 2018 Apr 23;18:1562–6. doi: 10.1016/j.dib.2018.04.061 (PMC5998173; doi:10.1016/j.dib.2018.04.061)
Supplement: Supplementary file 6 — Supplementary material [file mmc6.docx]

**Table S4.** *Mx* fold-change results per individual per tissue. Results are expressed as Normalized Relative Quantity (NRQ) and NRQ standard error (SE). Blanks occur when a particular tissue was not available for an individual due to labels peeling off tubes during transport in liquid nitrogen.

|  | **Spleen** | | **GI1** | | **GI2** | | **Colon** | |
| --- | --- | --- | --- | --- | --- | --- | --- | --- |
|  | **NRQ** | **SE** | **NRQ** | **SE** | **NRQ** | **SE** | **NRQ** | **SE** |
| **Sample 01** | 0.798 | 0.048 | 0.867 | 0.047 |  |  | 1.030 | 0.105 |
| **Sample 02** | 0.230 | 0.013 | 0.769 | 0.013 | 1.048 | 0.025 | 1.410 | 0.113 |
| **Sample 03** | 10.571 | 0.296 |  |  | 0.817 | 0.081 | 1.191 | 0.123 |
| **Sample 04** | 1.140 | 0.173 | 1.418 | 0.043 | 1.139 | 0.018 | 0.897 | 0.088 |
| **Sample 05** | 0.452 | 0.033 | 1.058 | 0.033 | 1.025 | 0.039 | 0.644 | 0.033 |
| **Sample 06** | 3.348 | 0.160 |  |  |  |  | 1.480 | 0.120 |
| **Sample 07** | 0.962 | 0.049 | 1.865 | 0.098 | 1.285 | 0.043 |  |  |
| **Sample 08** | 0.231 | 0.034 | 0.494 | 0.019 | 0.834 | 0.022 | 0.531 | 0.021 |
| **Sample 09** | 2.115 | 0.026 | 3.111 | 0.088 | 0.804 | 0.010 | 0.408 | 0.022 |
| **Sample 10** | 248.196 | 6.934 | 1.612 | 0.046 |  |  | 0.669 | 0.042 |
| **Sample 11** | 35.419 | 0.977 | 9.322 | 0.305 | 8.850 | 0.195 | 1.994 | 0.098 |
| **Sample 12** | 248.004 | 20.342 | 39.414 | 1.282 | 29.974 | 0.476 | 8.862 | 0.415 |
| **Sample 13** | 100.411 | 3.880 |  |  |  |  |  |  |
| **Sample 14** | 186.080 | 2.873 | 17.577 | 0.518 | 12.734 | 0.435 | 13.309 | 0.982 |
| **Sample 15** | 87.413 | 2.015 | 9.077 | 0.446 | 7.230 | 0.088 |  |  |
| **Sample 16** | 2.441 | 0.106 | 1.750 | 0.043 | 3.476 | 0.119 | 0.805 | 0.036 |
| **Sample 17** | 2.365 | 0.065 | 2.550 | 0.098 | 5.118 | 0.275 | 0.631 | 0.028 |
| **Sample 18** | 27.533 | 1.137 | 3.725 | 0.201 | 13.680 | 0.294 | 1.596 | 0.070 |
| **Sample 19** | 29.441 | 0.831 | 6.345 | 0.093 | 94.612 | 1.665 |  |  |
| **Sample 20** | 7.406 | 0.337 |  |  | 7.857 | 0.177 | 0.762 | 0.033 |
| **Sample 21** | 3.944 | 0.366 | 1.772 | 0.020 | 1.550 | 0.038 | 0.801 | 0.022 |
| **Sample 22** | 2.072 | 0.048 | 1.472 | 0.033 | 1.252 | 0.018 | 1.104 | 0.020 |
| **Sample 23** | 4.192 | 0.067 | 2.071 | 0.160 | 0.981 | 0.018 | 1.200 | 0.089 |
| **Sample 24** | 3.941 | 0.148 | 0.979 | 0.044 | 1.082 | 0.044 | 1.887 | 0.053 |
| **Sample 25** | 5.094 | 0.078 | 1.530 | 0.045 | 0.395 | 0.041 | 0.449 | 0.026 |
| **Sample 26** | 10.353 | 0.585 |  |  | 1.509 | 0.043 | 1.543 | 0.069 |
| **Sample 27** | 2.367 | 0.050 | 0.881 | 0.071 | 3.738 | 0.089 | 0.369 | 0.021 |
| **Sample 28** | 3.047 | 0.332 | 1.195 | 0.039 |  |  |  |  |
| **Sample 29** | 7.187 | 0.413 | 2.957 | 0.058 | 0.990 | 0.013 |  |  |
| **Sample 30** | 1.752 | 0.056 | 2.813 | 0.086 | 1.930 | 0.087 | 2.422 | 0.138 |
